# Tropical Diseases

**Published:** 1898-11-19

**Authors:** 


### The Hospital

A Journal of

The Medical Sciences and Hospital Administration.

Vol. XXV.—No. 634.] Edited by Sir Henry Burdett, K.C.B., and Solomon C. Smith, M.D., M.R.C.P. [Nov. 19, 1898.

### TROPICAL DISEASES.

The recent decision of the Colonial Office to take the initiative in providing instruction in tropical diseases for medical men who may hereafter be selected for employment in that department of the public service, and to throw open this instruction for the benefit of others who may proceed to the tropics in any private capacity, either as practitioners or as missionaries, may be said to mark a great step in the direction of acknowledging ministerial responsibility for the preservation of the health of the Queen Empress's subjects, whatever may be the colour of their skins, or whatever may be the portion of her Majesty's wide dominions in which their lot is cast, either as sojourners or as residents. The sagacity displayed by Mr. Chamberlain, to whom the measure may with something more than probability be referred, has been met with abundant public spirit by the governing body of the Seamen's Hospital; and it is hoped that the contemplated new buildings and other arrangements at that institution may be in complete working order before the close of the coming year. The Medical Council, when appealed to by the Colonial Office with regard to the question, was constrained to reply that, although lectures on tropical diseases were given at most of the metropolitan medical schools, yet that the calls made upon students for the acquisition of knowledge more immediately available in this country, and better calculated to "tell" in home examinations, were such as to place considerable difficulties in the way of any addition either to the compulsory curriculum or to the subjects in which a pass knowledge might reasonably be required as a qualification for practice. It is in complete accordance with this view that young medical men who accept certain responsibilities, which in their nature are mainly foreign to any which would devolve upon them in England, should undergo some special training after their general education has been rendered legally complete, and when the pressure of anxiety about the ordinary examinations has been removed. The value of the Seamen's Hospital as a field for such training can hardly be overrated, because the sick sailors who are received into its wards, and who will in most cases have been to all appearance in sound health when shipped at the commencement of a voyage, will not unfrequently present for observation those early symptoms with which, rather than with the later ones of the same affection, it is especially important that our Colonial medical officers should be conversant and prepared to cope. In this respect the hospital will have great advantages over Netley, where, as is well known, the admissions are chiefly of men in advanced stages of disease, who have already been under treatment, often for long periods, in military hospitals at the stations from which they have been sent home.

Nor is it less a triumph of Mr. Chamberlain's robust common sense that he should have declared the intention of the Government to promote and to subsidise research, and should have given evidence of his sincerity by the appointment of a Commission, the members of which have been selected and instructed in accordance with a scheme approved by the Royal Society, for the purpose of investigating the whole subject of tropical malaria. It is a disgrace to the practical common sense with which Englishmen are often credited that, in such matters, we should lag behind smaller, poorer, and generally less enlightened nations, and that our physicians should be compelled to seek from foreign countries information which they would be pre-eminently capable, if not hindered by artificial impediments, of obtaining for themselves. It is more than likely, when the Commission begins its labours, that some of the persons who conduct anti-vivisection societies may find it profitable to establish special branches, and to call for special subscriptions, for the rescue of the gentle and intelligent mosquito from the hands of barbarous persons who would interfere with its characteristic activities, or who might even carry cruelty so far as to observe the effects produced upon its system by compulsory variations of its accustomed diet, or even by complete deprivation of that vital fluid of the human system which may be presumed to stand to it somewhat in the relation of vintage port to the human connoisseur. Mr. Chamberlain's determination is the more to be commended because, as is well-known, the chief scientific want of England is that of positions which, together with freedom from anxiety about maintenance, would afford to competent workers leisure and opportunity for prolonged and difficult inquiries, either in the domain of physics or in that of biology. Such inquiries in this country cannot be conducted by anyone who is not possessed of sufficient private means to be independent of any necessity to earn; and even when completed they either bring no reward at all, or none which is in the least commensurate with their value to the community.

